# Genetic Diversity of *Brucella melitensis* in Kazakhstan in Relation to World-Wide Diversity

**DOI:** 10.3389/fmicb.2019.01897

**Published:** 2019-08-13

**Authors:** Elena Shevtsova, Gilles Vergnaud, Alexandr Shevtsov, Alexandr Shustov, Kalysh Berdimuratova, Kasim Mukanov, Marat Syzdykov, Andrey Kuznetsov, Larissa Lukhnova, Uinkul Izbanova, Maxim Filipenko, Yerlan Ramankulov

**Affiliations:** ^1^National Center for Biotechnology, Nur-Sultan, Kazakhstan; ^2^Institute for Integrative Biology of the Cell (I2BC), CEA, CNRS, Univ. Paris-Sud, Université Paris-Saclay, Gif-sur-Yvette, France; ^3^Kazakh Scientific Center for Quarantine and Zoonotic Diseases, Almaty, Kazakhstan; ^4^Institute of Chemical Biology and Fundamental Medicine, Novosibirsk, Russia; ^5^Synthetic Biology Department, Novosibirsk State University, Novosibirsk, Russia; ^6^School of Science and Technology, Nazarbayev University, Nur-Sultan, Kazakhstan

**Keywords:** *Brucella melitensis*, Kazakhstan, genotyping, genetic diversity, multiple-locus variable-number tandem repeat (VNTR) analysis (MLVA)

## Abstract

We describe the genetic diversity of 1327 *Brucella* strains from human patients in Kazakhstan using multiple-locus variable-number tandem repeat (VNTR) analysis (MLVA). All strains were assigned to the *Brucella melitensis* East Mediterranean group and clustered into 16 MLVA11 genotypes, nine of which are reported for the first time. MLVA11 genotype 116 predominates (86.8%) and is present all over Kazakhstan indicating existence and temporary preservation of a “founder effect” among *B. melitensis* strains circulating in Central Eurasia. The diversity pattern observed in humans is highly similar to the pattern previously reported in animals. The diversity observed by MLVA suggested that the epidemiological status of brucellosis in Kazakhstan is the result of the introduction of a few lineages, which have subsequently diversified at the most unstable tandem repeat loci. This investigation will allow to select the most relevant strains for testing these hypotheses via whole genome sequencing and to subsequently adjust the genotyping scheme to the Kazakhstan epidemiological situation.

## Introduction

Brucellosis is a zoonotic infection affecting many mammals including marine species as well as humans. Infection is endemic in many countries of the world, the etiological agent being a Gram-negative bacteria of the genus *Brucella*. Among the 12 species currently proposed in this genus, *Brucella melitensis*, *B. abortus*, and *B. suis* are highly dangerous for humans and cause disease with severe complications and chronic process (Meyer, 1990; Godfroid et al., 2011). Rare cases of infection of people with other *Brucella* species are also recorded (Ficht, 2010). Despite its low mortality rates, brucellosis is a very important public health problem in Kazakhstan. Husbandry suffer direct economic losses from brucellosis due to reduced productivity, culling of livestock and costs of associated measures. In spite of the control strategies, brucellosis remains a major economic problem for agriculture, with an annual direct cost of more than $45 million and a social problem in health care with an average disability-adjusted life year of 0.5 per case (Charypkhan et al., 2019). In addition, aerosol transmission and low infectious doses (10–100 bacteria) potentially allow using *Brucella* in acts of bioterrorism (Pappas et al., 2006b; Neubauer, 2010; Doganay and Doganay, 2013).

Control of animal infections is essential to prevent human infections. In developed countries, epidemiological measures were successfully undertaken to eliminate brucellosis in domestic animals, resulting in that all human cases are recorded among migrants, or associated with traveling to endemic areas, consumption of imported products, and in rare cases contacts with wild animals (Gwida et al., 2012). On the contrary, in many countries of the Mediterranean region, Southern and Central America, Africa, Asia, Arabian Peninsula, Indian subcontinent, Eastern Europe, and Middle East, the brucellosis epidemiological situation remains complex allowing to describe it as a continuing epidemic (Nicoletti, 2010).

Given the zoonotic nature of brucellosis, identification of sources of the infection and tracking of transmission paths are of high importance for epidemiologic surveillance. Reliable discrimination among *Brucella* species and biovars are important for treatment, due to differences in pathogenicity between various *Brucella*. DNA-based genotyping methods are preferred because molecular methods minimize the risks of infection of laboratory personnel and phenotypic methods have a much lower discriminatory power. For *Brucella*, a number of genotyping methods have been proposed including AMOS-PCR, Bruce-ladder, tandem-repeats polymorphism [multiple-locus VNTR analysis (MLVA)] and whole genome SNP analysis (wgSNP). wgSNP analysis is by far the most powerful and robust approach, but its current cost does not allow yet to use it as a routine first line assay (Ledwaba et al., 2019). MLVA typing is a compromise in terms of discriminatory power, cost and speed to provide an overview of genetic diversity and assist the selection of strains for whole genome sequencing (Scholz and Vergnaud, 2013). Availability of freely accessible databases with MLVA genotypes allow to track the spreading of specific *Brucella* biovars globally (Kattar et al., 2008; Kilic et al., 2011; Ferreira et al., 2012; Vergnaud et al., 2018). Fundamental for this work is genotyping and consolidation of data from all endemic regions.

Kazakhstan is located in the center of Eurasia. It covers a territory of 2.725 million km^2^ for 18 million inhabitants. The country shares land borders with Russia, China, Kyrgyzstan, Uzbekistan, Turkmenistan. With regard to brucellosis, Kazakhstan is a hyperendemic area considering the very high incidence rates in the human population and farm animals (Pappas et al., 2006a). According to evaluations in the Former Soviet Union (FSU), more than 40% of newly diagnosed brucellosis cases are from Kazakhstan (Studentsov, 1975). From 1999 to 2016, 38,557 new cases of human brucellosis were recorded, with annual counts of cases ranging from 1443 (2014) to 3596 (2004) (Syzdykov et al., 2013; Shevtsova et al., 2016; Daugaliyeva et al., 2018). Within these 18 years, the majority of cases (89.5%) occurred in southern and eastern Kazakhstan (Almaty, Zhambyl, Kyzylorda, East-Kazakhstan, and South-Kazakhstan regions). 75% of the cases of newly diagnosed brucellosis were in the age group 10–39 years, and 85.8% were rural residents. Most strains recovered from patients were identified as *B. melitensis* (Mizanbayeva et al., 2009). The epidemiological situation correlates with occurrence of brucellosis in small ruminants, e.g., the mentioned geographic regions hold more than 70% of all identified seropositive animals among small ruminants (data collected during 2008–2015) (Espembetov et al., 2017). From 2006 to 2015, the average level of seropositive small ruminants ranged from 0.15 to 0.3% with an increase in 2008 (0.65%) and 2013 (0.5%). The fluctuation from 0.01 to 2.5% of the percentage of seropositive animals at the district level is uncorrelated with the level of infection in humans (Daugaliyeva et al., 2018; Charypkhan et al., 2019). In cattle, the incidence of seropositive animals varied from 0.3 in 2006 to 0.6% in 2013–2015. The high incidence of brucellosis reported in cattle during 2009–2011 (1.85%) is most probably an artifact originating from a change in diagnostic procedures used in Kazakhstan. During this period, all cattle was screened using only an indirect enzyme-linked immunosorbent assay (iELISA) and classical methods such as the rose Bengal test (RBT), serum agglutination test (SAT), and complement fixation test (CFT) were not used for confirmation of the brucellosis diagnosis, until the low specificity of the iELISA became evident. Starting from 2012, the classical methods were again made compulsory for confirmation of brucellosis diagnosis which resulted in reduction of the reported incidence (Espembetov et al., 2017). In addition to the main reservoir hosts, brucellosis caused by *B. melitensis* or *B. abortus* is registered in camels, horses, and dogs (Zheludkov and Tsirelson, 2010). *Brucella* antibodies have been detected in more than 60 wild mammals (Shevtsova et al., 2016).

The high morbidity and large territory of Kazakhstan make it essential to describe the genetic diversity and distribution of genotypes among circulating strains of *Brucella* in order to support efforts for disease control. Previous reports have described the genetic diversity of *Brucella* isolated from animals in Kazakhstan (Shevtsov et al., 2015; Shevtsova et al., 2016; Daugaliyeva et al., 2018; Yespembetov et al., 2019). The purpose of the present study was to determine the genetic diversity of the strains infecting humans at the whole country scale.

## Materials and Methods

### Ethics Statement

A formal institutional ethical review process and approval was not required for this study as all strains investigated here were collected as a part of standard clinical investigation of patients with suspected brucellosis and the strains were anonymized. All *Brucella* strains used in this study were obtained from the collection maintained by the Masgut Aykimbayev Kazakh Scientific Center of Quarantine and Zoonotic Diseases (KSCQZD) in accordance to Kazakhstan regulations.

### Clinical Strains Characterization

A total of 1383 *Brucella* strains were isolated from patients seropositive to brucellosis antigen (agglutination test 1:200 and higher) during 2015–2016 in 13 regional infectious diseases hospitals. The hemocultures were produced by the two-phase method proposed by Castaneda (Castaneda, 1947; Yagupsky, 1999). All strains were Gram negative, agglutinated with polyvalent brucellosis serum, had oxidase and catalase activity, did not produce H_2_S, synthesized urease, and were capable of growing in plain atmospheric conditions. Biotyping was performed by KSCQZD by routine test (Al Dahouk et al., 2003).

### DNA Preparation, Genotyping, and Data Analysis

Bacterial culture was scrapped from the surface of the solid agar medium, resuspended in 500 μl *Tris* 10 mM EDTA 1 mM pH 8 (TE) buffer and inactivated by adding an equal volume of chloroform. DNA was isolated using the QIAamp DNA Mini Kit (Qiagen, United States). Species identification was confirmed by the multiplex PCR “Bruce-ladder” assay (Lopez-Goni et al., 2008). The 16S rDNA fragment was sequenced to confirm taxonomic identification of the genus and to exclude presence of extraneous microflora.

Markers proposed by Le Flèche et al. (2006) and Al Dahouk et al. (2007) were used for MLVA. The 16 loci are commonly divided into three panels. The first panel (also called MLVA8) consists of the eight tandem repeat loci with the largest repeat units, Bruce06, Bruce08, Bruce12, Bruce42, Bruce43, Bruce45, and Bruce55. The eight other tandem repeats have smaller repeat units up to eight base-pairs. The second panel (panel 2A) includes Bruce18, Bruce19, and Bruce21. Panel 2B includes the last five loci: Bruce04, Bruce07, Bruce09, Bruce16, and Bruce30. The combination of panels MLVA8 and 2A is called MLVA11, while the combination of all three panels (16 loci) is designated MLVA16. The MLVA11 panel allows for species identification in the *Brucella* genus and grouping of strains in accordance to the global geographic distribution. The panel 2B loci are highly discriminatory and their combination with MLVA11 is used in tracking local outbreaks. New variable-number tandem repeat (VNTR) alleles were confirmed by sequencing the PCR product.

Primers for multiplex PCR and their combinations were used as previously described (Garofolo et al., 2013; Shevtsov et al., 2015). PCR amplification products were diluted 1/70, and aliquots (3 μl) of the diluted samples were run on a capillary electrophoresis machine (DNA Analyzer 3730xl, Applied Biosystems, Japan), using the POP7 polymer and LIZ 1200 size standard. Size analysis of VNTR repeats was performed using GeneMapper 4.1 (Applied Biosystems).

To visualize clustering relations, maximum parsimony analysis dendrograms were constructed using BioNumerics 7.6 (Applied Maths, Sint-Martens-Latem, Belgium). The Hunter and Gaston diversity index (HGDI) was used to describe discriminatory capacity of each locus, as well as of the panels MLVA8, MLVA11, and MLVA16 (Hunter and Gaston, 1988). Computations were performed using the Internet resources at www.hpa-bioinfotools.org.uk/cgi-bin/DICI/DICI.pl. Geographic coordinates (longitude, latitude) were deduced from available geographic origin data using the BioNumerics geographical plugin. The geographic location map was drawn using QGIS 3.4.8-Madeira (QGIS Geographic Information System. Open Source Geospatial Foundation)^1^.

## Results

A total of 1383 strains *Brucella* collected from 13 regions of Kazakhstan were studied. All strains could be assigned to the *Brucella* genus based on analysis of a fragment of the nucleotide sequence of 16S rDNA and to *B. melitensis* according to the Bruce-ladder profile. The biovar was established for 500 strains. Ninety-five (19%) were assigned to biovar 1, six (1.2%) to biovar 2 and 399 (79.8%) were assigned to biovar 3 (Supplementary Table S1). The 16 VNTR loci constituting the MLVA assay were successfully amplified in all DNAs. Five samples showed a mixed pattern for the moderately variable Bruce11 locus. Twenty-seven samples demonstrated a mixed pattern in one of the highly variable loci constituting panel 2A and 2B: Bruce16 (15 isolates), Bruce18 (four isolates), Bruce30 (three isolates), Bruce19 and Bruce07 (two isolates each), Bruce04 (one isolate). Twenty-four additional isolates showed mixed patterns at more than two highly variable loci, in agreement with previous observations (Whatmore et al., 2006; Maquart et al., 2009). The 56 samples showing mixed patterns were excluded from further analyses. A new Bruce43 allele (206 bp, called 4) was observed in strains NCB#h-2315, NCB#h-3375 and NCB#h-3362. Two new Bruce19 alleles (106 bp, called 17 and 208 bp called 51) were observed in three strains (NCB#h-3378, NCB#h-3379, and NCB#h-3409) and one strain (NCB#h-2223), respectively. These new alleles were confirmed by sequencing of the PCR products.

The HGDI index of the 16 VNTR loci and of panels MLVA8, MLVA11, MLVA16 are indicated in Table 1. In panel MLVA11, only 5 among the 11 loci were polymorphic in the present collection. In panel 2B, all five loci were variable, with loci Bruce07 and Bruce09 being the least variable. The HGDI for MLVA8 and MLVA11 were 0.163 and 0.242, respectively. The HGDI for MLVA16 was 0.989. In countries with more than 50 entries in MLVA bank, variable HGDI levels could be observed (Table 2). In Asian countries including Kazakhstan, panel2B loci Bruce07 and Bruce09 consistently showed low HGDI values. Locus Bruce30 is invariable in strains sampled from Africa and South America, and moderately variable in Italy. In most European countries, all loci of the panel 2B are highly discriminatory.

**TABLE 1:** Hunter and Gaston diversity index (HGDI) for loci and MLVA panels for 1327 *B. melitensis* strains from human patients in Kazakhstan.

**Locus**	**Diversity index**	**Confidence interval**	**Number of genotypes observed**	Max(pi)^*^
Bruce06	0.000	0.000–0.006	1	1.000
Bruce08	0.025	0.020–0.031	3	0.987
Bruce11	0.000	0.000–0.006	1	1.000
Bruce12	0.000	0.000–0.006	1	1.000
Bruce42	0.000	0.000–0.006	1	1.000
Bruce43	0.142	0.130–0.153	4	0.924
Bruce45	0.000	0.000–0.006	1	1.000
Bruce55	0.000	0.000–0.006	1	1.000
MLVA8	0.163	0.150–0.175	7	0.913
Bruce18	0.049	0.041–0.056	3	0.975
Bruce19	0.052	0.044–0.059	6	0.974
Bruce21	0.000	0.000–0.006	1	1.000
MLVA11	0.242	0.228–0.256	16	0.868
Bruce04	0.770	0.764–0.775	11	0.349
Bruce07	0.292	0.277–0.306	6	0.835
Bruce09	0.016	0.012–0.021	4	0.992
Bruce16	0.786	0.782–0.791	12	0.320
Bruce30	0.632	0.624–0.640	6	0.503
MLVA16	0.989	0.988–0.989	310	0.050

^*^Max(pi) = fraction of samples that have the most frequent repeat number in this locus (range 0.0–1.0).

**TABLE 2:** HGDI for the panel 2B for East Mediterranean *B. melitensis* from various countries.

**Country**	**Bruce04**	**Bruce07**	**Bruce09**	**Bruce16**	**Bruce30**	**Number of strains**
Kazakhstan	0.770	0.292	0.016	0.786	0.632	1327
France	0.851	0.720	0.858	0.880	0.540	479
Spain	0.807	0.778	0.371	0.814	0.680	403
China	0.750	0.114	0.109	0.834	0.754	303
Turkey	0.762	0.487	0.195	0.782	0.737	233
Italy	0.798	0.784	0.894	0.817	0.070	225
Portugal	0.760	0.737	0.321	0.777	0.451	149
Peru	0	0.164	0.689	0.18	0	114
Greece	0.774	0.279	0.57	0.778	0.75	78

**FIGURE 1:**
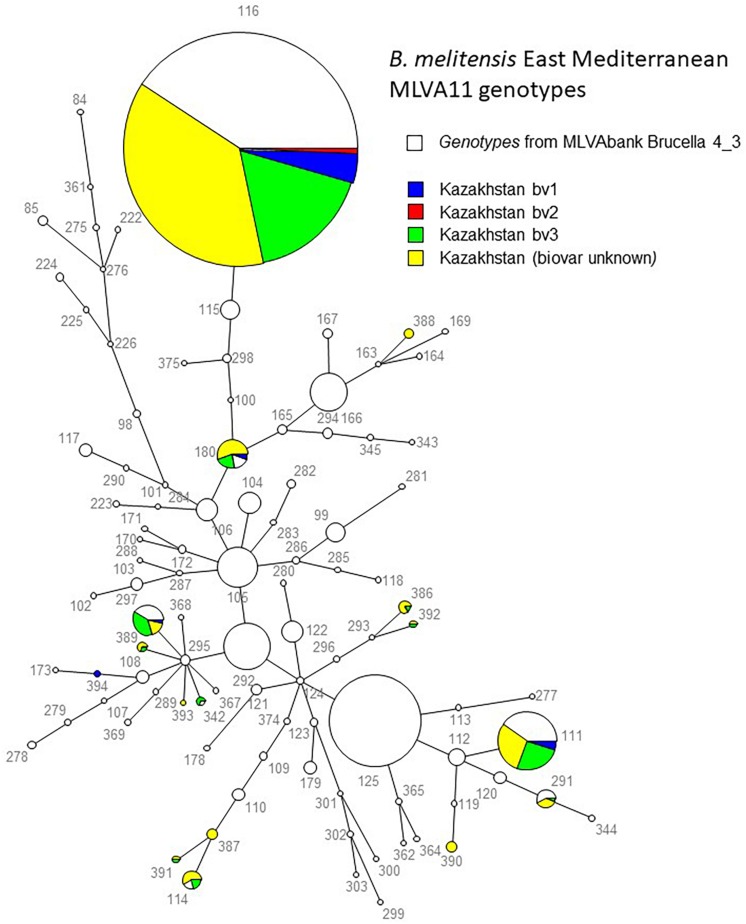
MLVA11 in 2900 East Mediterranean *B. melitensis* entries including 1580 from *Brucella* MLVAbank version 4.3. The 1327 strains from the present study are colored. The biovar assignment is indicated when known. The numbers are the MLVA11 genotypes. The genotypes are connected by branches with a length of one or two corresponding to the categorical distance between them.

**FIGURE 2:**
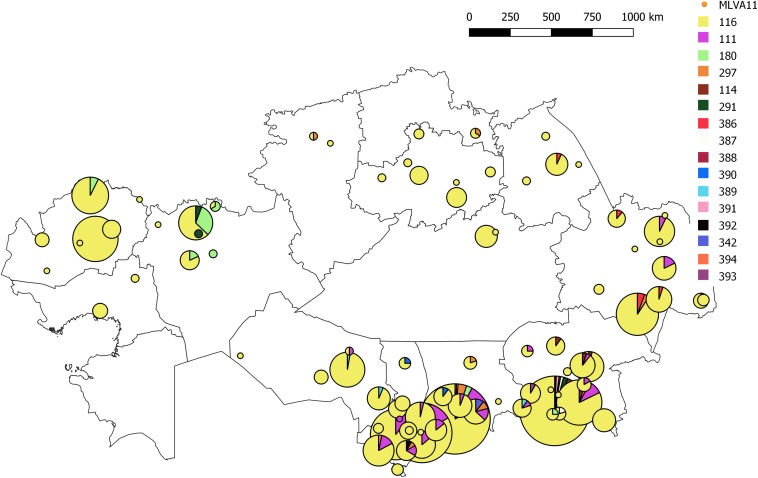
Map of Kazakhstan showing the distribution of *B. melitensis* MLVA11 genotypes of the 1327 strains from human patients. The color code reflects the MLVA11 genotype. The number of strains assigned to a given genotype is indicated in detail in Supplementary Table S2.

All *B. melitensis* isolates isolated in Kazakhstan cluster within the East Mediterranean group congruently defined by MLVA, MLST and wgSNP (Whatmore et al., 2016; Vergnaud et al., 2018). In agreement with previous observations, all three *B. melitensis* biovars are observed within the East Mediterranean group. In the whole *Brucella* genus, 383 MLVA11 genotypes are defined, 100 of which belong to the East Mediterranean group (Figure 1). The 1327 *B. melitensis* strains from human patients isolated in Kazakhstan were clustered into 16 genotypes, nine of which numbered 386–394 have not been reported previously (Table 3). The most prevalent, genotype 116, was found in 86.8% of samples, genotype 111 in 6.03%, genotype 180 in 2.18%, and genotype 297 in 1.51%. The 12 other genotypes are rare (<1%). Genotype 116 is widespread in Kazakhstan whereas genotype 111 is detected in the hyperendemic areas in the southern part of Kazakhstan. The other genotypes have small prevalence in Kazakhstan and tend to be present in Southern Kazakhstan although genotypes 180 and 297 were also recovered in Northern Kazakhstan (Figure 2 and Supplementary Table S2).

**TABLE 3:** Distribution of genotypes by MLVA8 and MLVA11 in Kazakhstan.

**MLVA11 profile**	**MLVA8 genotype (number of strains/%)**	**MLVA11 genotype (number of isolates/%)**
**1-5-3-13-2-2-3-2**-4-41-8^*^	42 (1211/91.25)	116(1152/86.8)
**1-5-3-13-2-2-3-2**-5-41-8		180(29/2.18)
**1-5-3-13-2-2-3-2**-4-46-8		297(20/1.51)
**1-5-3-13-2-2-3-2**-3-41-8		342(2/0.15)
**1-5-3-13-2-2-3-2**-4-17-8		388(4/0.3)†
**1-5-3-13-2-2-3-2**-4-36-8		389/(3/0.23)†
**1-5-3-13-2-2-3-2**-4-51-8		393(1/0.15)†
**1-5-3-13-2-3-3-2**-4-41-8	63 (89/6.71)	111(80/6.03)
**1-5-3-13-2-3-3-2**-4-46-8		386(6/0.45)†
**1-5-3-13-2-3-3-2**-4-36-8		392(2/0.15)†
**1-5-3-13-2-3-3-2**-4-43-8		394(1/0.15)†
**1-3-3-13-2-2-3-2**-4-41-8	62 (11/0.83)	114(11/0.83)
**1-5-3-13-2-1-3-2**-4-41-8	114 (7/0.53)	291(7/0.53)
**1-4-3-13-2-2-3-2**-4-41-8	115 (4/0.3)	387(4/0.3)†
**1-5-3-13-2-4-3-2**-4-41-8	196 (3/0.23) †	390(3/0.23)†
**1-4-3-13-2-3-3-2**-4-41-8	197(2/0.15) †	391(2/0.15)†

^*^MLVA8 in bold.†New genotype.

**FIGURE 3:**
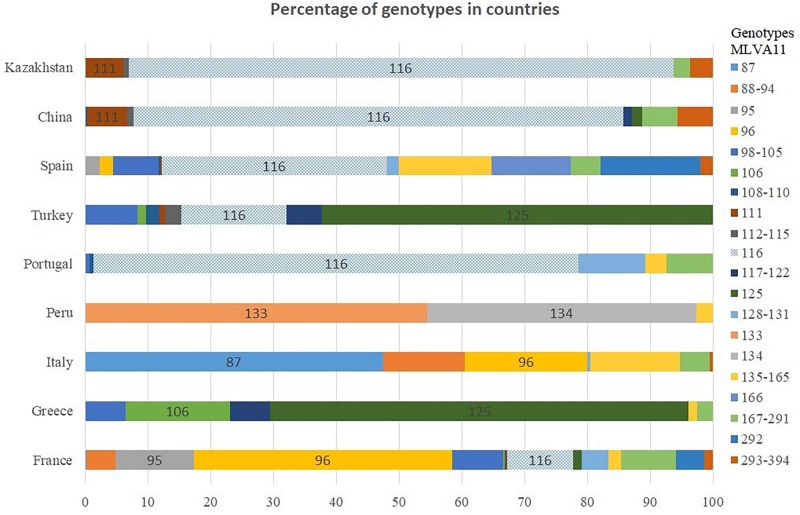
Distribution of *B. melitensis* MLVA11 genotypes by country.

The MLVA genotypes deposited in *Brucella* MLVAbank version 4_3 can be used to compare the incidence of these genotypes in different regions of the world. Genotypes 116 and 111 represent 50 and 3.2%, respectively of the more than 1500 East Mediterranean strains present in MLVAbank (Supplementary Table S1). In Portugal and China, genotype 116 accounts for more than 77% of cases, 37% in Spain and the prevalence descends further in Turkey (16%) and France (10%) (Figure 3). Genotype 116 is predominant in Asian countries, as is evidenced by the identification of only this genotype in *B. melitensis* isolates from India and Mongolia, although the sampled populations were small. Genotype 111 is also relatively frequent in Asia. The rare genotypes 291 and 387 have been found only in Kazakhstan and China, whereas genotype 114 was found only in Kazakhstan and Turkey. Genotype 125, which is the second most frequent (18.5%) in East Mediterranean strains represented in MLVAbank and is the most prevalent genotype in Greece and Turkey, was not detected in Kazakhstan.

**FIGURE 4:**
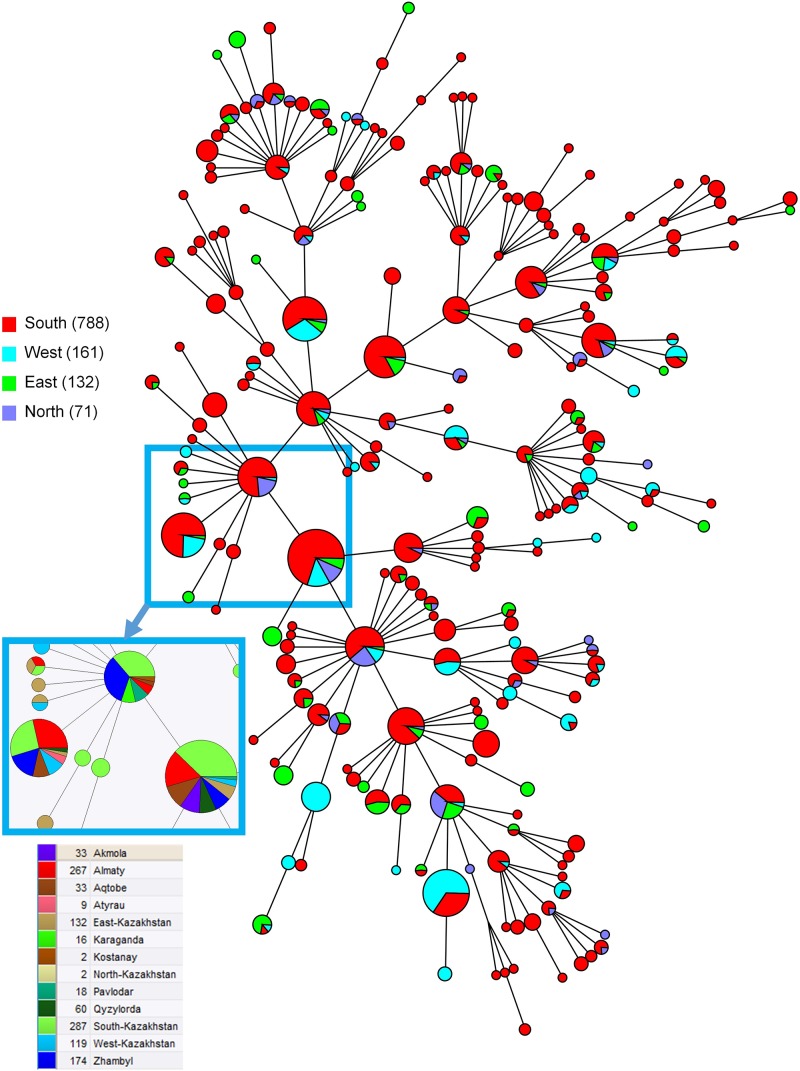
Maximum parsimony analysis using MLVA16 for 1152 genotype 116 *B. melitensis* strains isolated from humans in Kazakhstan. The color code reflect the geographic origin of the strains (South: Almaty, Qyzylorda, Zhambyl, South-Kazakhstan; West: Aqtobe, Atyrau, West Kazakhstan; East: East Kazakhstan; North: Akmola, Kostanay, Karaganda, Pavlodar, North Kazakhstan). Inset: color code by region for a subset of the most frequent MLVA16 genotypes showing their wide geographic spreading. Branch lengths correspond to one up to two differences among the 16 values constituting the MLVA16 genotype.

**FIGURE 5:**
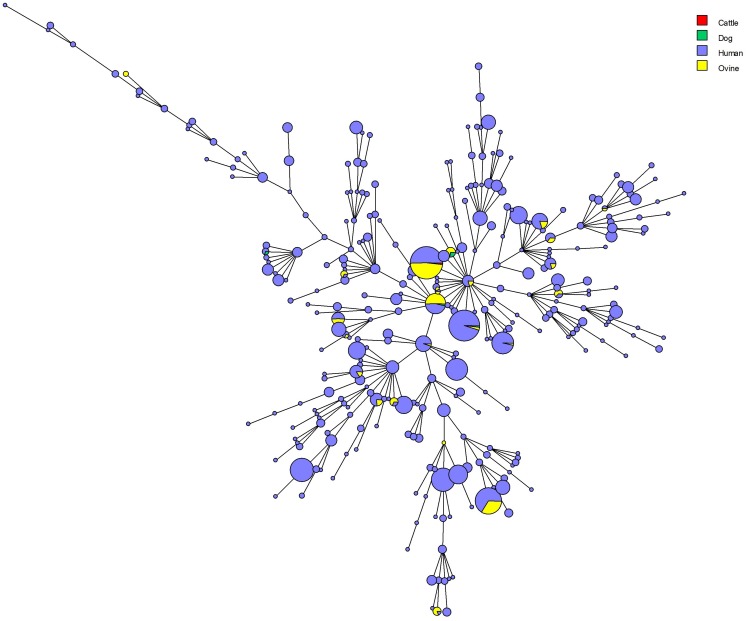
Maximum parsimony analysis using MLVA16 data from 1455 *B. melitensis* strains isolated from humans and animals in Kazakhstan. The color code reflects the host origin (cattle: 1 strain, genotype 116; dog: 5 strains, 2 belong to genotype 111, 3 to genotype 116; human: 1327 strains; sheep: 122 strains).

The MLVA16 clustered the 1327 strains into 310 genotypes, 131 of which were represented by a single isolate, 53 by two isolates, and 33 by three isolates. The most prevalent genotype was found in 66 strains (Figure 4). Among the 29 most frequent genotypes (found in 10 or more isolates) one genotype with 17 isolates was restricted to one geographic region (West Kazakhstan), whereas other most prevalent genotypes were present in a wider area. Among 61 medium-prevalence genotypes (e.g., found in four to nine samples), geographic linkage was established for ten genotypes: four genotypes are confined to Almaty region, five genotypes to East Kazakhstan, and one to West Kazakhstan. Figure 5 shows the diversity of genotypes observed in humans, together with diversity previoulsy reported in animals (Shevtsov et al., 2015).

**FIGURE 6:**
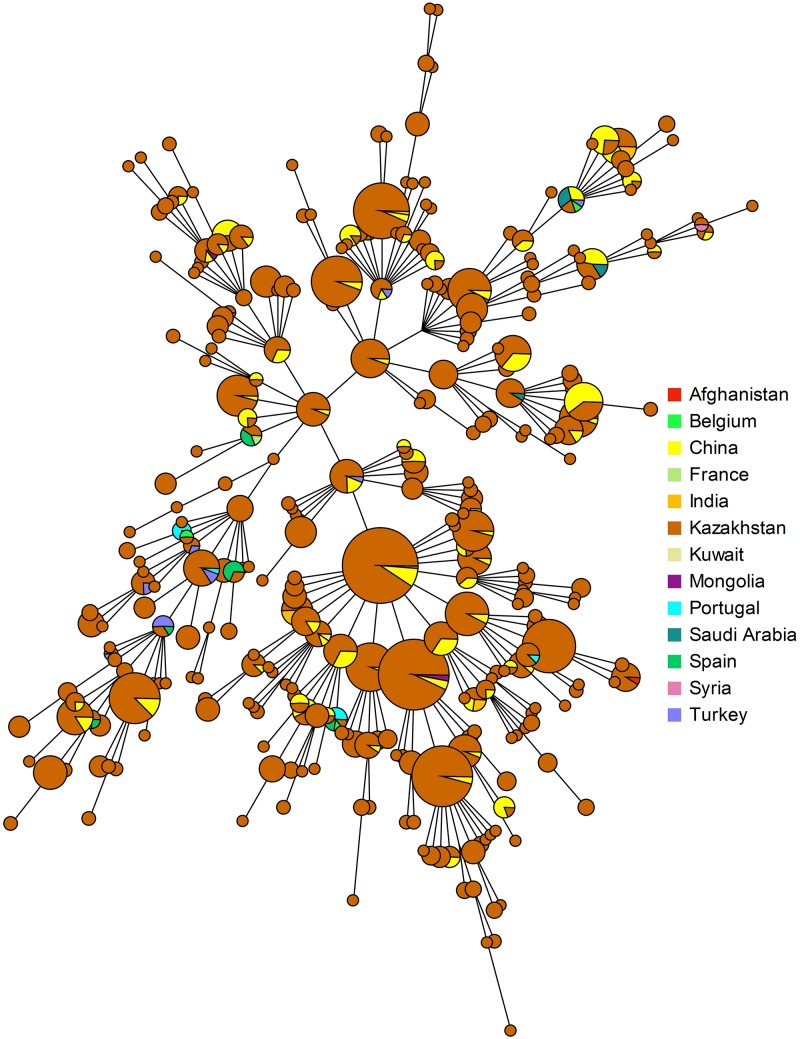
Geographic origin of strains present in MLVAbank Brucella_4_3 and showing an MLVA16 genotype present in Kazakhstan.

Clustering analysis using all 16 VNTR loci (MLVA16) was conducted including *B. melitensis* East Mediterranean data from MLVAbank. Shared genotypes were found for 83 (among 313) genotypes representing more than 57% of the isolates (Figure 6). The highest number of shared genotypes was found for strains circulating in China and Kazakhstan. Fifty-four MLVA16 genotypes are common between 530 isolates from Kazakhstan and 172 strains from China. Shared genotypes were also observed with *Brucella* strains isolated in other Asian and European countries. Eleven MLVA16 genotypes were present in a large geographic space, including Kazakhstan (242 isolates), China and other Asian and Middle-East countries (Turkey, Saudi Arabia, India, Mongolia, and Kuwait). Identical MLVA16 genotypes were observed among strains from Kazakhstan and Syria (one genotype, one strain for each country), Kazakhstan and Afghanistan (1 genotype, 10 and 1 strain(s), respectively), Kazakhstan and India (one genotype, one strain from each country), Kazakhstan and Saudi Arabia (1 genotype, 10 and 1 strain(s), respectively), Kazakhstan and Turkey (5 genotypes, 26 and 7 strains, respectively). Ten MLVA16 genotypes from Kazakhstan (comprising 37 strains) were found in European countries: Spain (6 genotypes, 11 strains), Portugal (4 genotypes, 6 strains), and France and Belgium (2 genotypes, 2 strains).

## Discussion

We report here the largest investigation of the genetic diversity of *Brucella* in a single country. More than 1300 strains were collected from human patients and genotyped using tandem repeats polymorphism. Despite the fact that both *B. melitensis* and *B. abortus* are circulating among farm animals, and that the live vaccines from strains Rev1 (*B. melitensis* “Americas”), S19, SR82, and RB51 (*B. abortus*) are used in Kazakhstan, all strains from humans in this study were identified as *B. melitensis* “East-Mediterranean” (Shevtsova et al., 2016; Daugaliyeva et al., 2018). *B. melitensis* biovar 3 is predominant in this collection (79.8%), whereas biovars 1 and 2 account for 19 and 1.2%, respectively. Predominance of biovar 3 is typical in neighboring countries as well as in the whole Eurasian region (FAO, 2010; Liu et al., 2017). The results obtained show a very good correlation between the genotypes present in livestock in Kazakhstan and the genotypes observed in humans, as expected if infections occurred locally. Brucellosis is a typical zoonotic disease and better monitoring of *Brucella* circulation in animal populations will hopefully contribute to the successful control of human brucellosis.

Multiple-locus VNTR analysis genotyping assigned all strains to the *B. melitensis* “East-Mediterranean” group with predominance of the MLVA11 genotypes 116 and 111 (86.8 and 6.03%, respectively). The recovery of genotype 116 across most of Kazakhstan may be explained by extensive movement of livestock between regions by nomadic herders. The nomadic style is a historic way of livestock husbandry in Kazakhstan and was preserved until the beginning of the 20th century. Nomadism implies regular movement of herds to new pastures many times per year (Mukhatova, 2014). Similarly, uncontrolled movement of livestock is described as the main source for geographic propagation of the infection (Syzdykov et al., 2014).

In contrast, genotype 111 is almost exclusively observed in South Kazakhstan, Almaty, Zhambyl, East-Kazakhstan, and Qyzylorda regions. These regions suffer from the most aggravated epidemiological situation. They account for 70% of infected sheep in Kazakhstan (during 5-year observation, 2011–2015), and hold 67% of small ruminants population in the country (Syzdykov et al., 2014; Espembetov et al., 2017). Two of the mentioned Kazakhstan’s regions share long borders with China. Information is lacking on genetic diversity in the other countries bordering Kazakhstan along the southwestern border.

The distribution of MLVA genotypes in Kazakhstan circulating in animals and humans closely replicated the genotypic distribution in the brucellosis-hyperendemic regions of Northern China, and more than 50% of the isolates from Kazakhstan have analogs among strains from China. This similarity supposedly reflects a common circulation in the neighboring countries, although it is not clear if the cross-border circulation still exist. MLVA typing does not allow establishing at what historical epoch the genotypic structure shaped into its current form. Trade ties and livestock exchange between the countries have long history, archeological finds indicate trade relations between the nomadic peoples who inhabited Central Asia and China long before the Common Era (CE) (Dicosmo, 1996). In addition, 4500 years ago, seasonal nomadic pastoralist routes were formed from modern southern Kazakhstan to Xinjiang Uygur Autonomous Region, covering the territory of more than 70% of the high-mountain route of the silk road (Frachetti et al., 2017). Cross-border nomadic pastoralism played an important role in the functioning of the Silk Road until the 15th century CE (Hermes et al., 2018). Trade relations between China and nomadic pastoralists of Kazakhstan continued until the formation of the Soviet Union. During the Soviet period, cattle was run from Mongolia to the Western parts of the Soviet Union passing through Kazakhstan (Kuznetsov, 1962). In modern history there is no trade in live cattle between Kazakhstan and China, but actively developing trade in livestock products.

## Conclusion

Multiple-locus VNTR analysis genotyping constitute a convenient medium-resolution classification assay for large-scale investigations. Follow-up studies based on whole genome sequencing of selected strains will be necessary to more precisely decipher the dynamics of strain circulation both within Kazakhstan and with Kazakhstan’s neighboring countries. Such studies will also permit to design new low-cost genotyping assays tailored for Central Asia. These assays may be based on a combination of selected tandem repeats including currently unused loci and of key SNPs.

## Data Availability

The datasets generated for this study can be found in http://microbesgenotyping.i2bc.paris-saclay.fr.

## Author Contributions

GV edited the original manuscript. AShe conceptualized and designed the experiments. AShe, ES, and GV analyzed the data. ES, AShe, AShu, KB, KM, MF, and YR were involved in the genotyping and wrote the manuscript. MS, AK, LL, and UI did bacteriological researches and wrote the manuscript. All authors have read and approved the final version of the manuscript.

## Conflict of Interest Statement

The authors declare that the research was conducted in the absence of any commercial or financial relationships that could be construed as a potential conflict of interest.

## Footnotes

1 http://qgis.org
